# 3.0-Tesla MRI Observation at Return to Play After Hamstring Injuries

**DOI:** 10.1097/JSM.0000000000001289

**Published:** 2024-11-20

**Authors:** Muhammad Ikhwan Zein, Gustaaf Reurink, Jozef J. M. Suskens, Jithsa R. C. Monte, Frank F. Smithuis, Stan Buckens, Mario Maas, Johannes L. Tol

**Affiliations:** * Department of Orthopedic Surgery and Sports Medicine, Amterdam UMC Location University of Amsterdam, Amsterdam, the Netherlands;; † Amsterdam Movement Sciences, Sports, Amsterdam, the Netherlands;; ‡ Faculty of Sports Science, Universitas Negeri Yogyakarta, Depok, Indonesia;; § Academic Center for Evidence Based Sports Medicine (ACES), Amsterdam, the Netherlands;; ¶ AMC/VUmc IOC Research Center, Amsterdam Collaboration on Health & Safety in Sports (ACHSS), Amsterdam, the Netherlands;; ‖ Department of Sports Medicine, The Sports Physician Group, OLVG Hospital, Amsterdam, the Netherlands;; ** Department of Radiology and Nuclear Medicine, Amsterdam UMC Location University of Amsterdam, Amsterdam, the Netherlands;; †† Department of Radiology and Nuclear Medicine, Radboud University Medical Center, Nijmegen, the Netherlands; and; ‡‡ Aspetar, Orthopedic and Sports Medicine Hospital, Doha, Qatar.

**Keywords:** hamstring injuries, MRI, muscle injuries

## Abstract

**Objective:**

To describe 3.0-Tesla (T) magnetic resonance imaging (MRI) findings of hamstring muscles in clinically recovered athletes who were cleared for return to play (RTP).

**Design:**

Prospective observational study.

**Setting:**

Hospital.

**Participants:**

Athletes (amateur and professional) who were cleared for RTP after hamstring injury. Fifty-eight participants were included in the analysis.

**Independent Variables:**

3-T MRI at baseline (within 7 days from initial injury) and MRI at RTP (within 10 days of RTP).

**Main Outcome Measures:**

Injury location, grade of injury (modified Peetrons and British Athletics Muscle Injury Classification/BAMIC), presence and the extent of intramuscular signal abnormality, intramuscular tendon disruption, and thickness. Reinjuries within 1 year of RTP were recorded.

**Results:**

Magnetic resonance images at RTP showed that 55 (95%) participants had intramuscular increased signal intensity (edema) and 44 (76%) participants had intramuscular abnormal low-signal intensity (suggesting fibrosis) on MRI. There was an overall reduction of injury grades according to the modified Peetrons and BAMIC classification at initial injury to RTP. Three (5%) participants had no abnormal signal intensities (grade 0 or grade 0A) on MRI at RTP. Intramuscular tendon disruption, waviness, and tendon thickening were present at RTP in, respectively, 22 (38%), 15 (26%), and 36 (62%). We recorded 3 (5%) reinjuries.

**Conclusions:**

At RTP, 3.0-T MRI shows high percentages of MRI abnormalities (edema, fibrosis, and intramuscular tendon disruption and thickening). We conclude that complete normalization of 3.0-T MRI is not expected for RTP decision after a hamstring injury. Its possible association with reinjury risk has to be determined in larger cohorts.

## Abstract 2

Supplemental Digital Content is Available in the Text.

## INTRODUCTION

Hamstring injury is the most common muscle injury in sports with an associated high reinjury rate (13.9%-63.3%)^1^ and often causes a prolonged recovery period.^2–8^ More than 50% of these reinjuries occur within 4 weeks after return to play (RTP).^5,8^ Premature RTP is suggested as one of the factors of the high early reinjury rate.^1,9^

Two Delphi consensus studies on RTP after hamstring injury have suggested several RTP criteria, namely absence of pain, confidence and psychological readiness, functional performance including sprint ability, and full flexibility and hamstring strength.^10,11^ In addition to these clinical factors, magnetic resonance imaging (MRI) is used in the management of hamstring injuries. Several studies suggest that MRI might have a potential role in assisting RTP decisions, that is, to predict a time frame for RTP based on injury characteristic and grading system.^5,12–16^

Prospective studies by Silder et al^17^ and Sanfilippo et al^18^ in 21 and 25 acute hamstring injuries found that MRI at RTP showed signs of edema in 100% and 20.4% of the subjects, respectively. In our previous observational study in 53 participants, 89% of the clinically recovered hamstring injuries showed edema, indicating that normalization of edema on MRI is not required for successful RTP.^19^ Our other study also showed that the presence and extent of abnormally low signal intensity in the intramuscular tissue, suggestive of fibrosis, were present in 38% of MRIs at RTP.^20^ There was no association between fibrosis on MRI and the reinjury risk.^20^

These previous studies on MRI findings at RTP had several limitations. First, these studies were performed on 1.5-T.^17–20^ Currently, 3.0-T is the preferred standard and provides higher resolution, and produces more detailed images.^21^ We expect that 3.0-T MRI is more sensitive in detecting any structural damage of the tissue and thereby increasing the percentage of edema and fibrosis formation at RTP compared with our previous studies. Second, we did not describe intramuscular tendon injury in the previous studies. In recent years, several studies found (intramuscular) tendon involvement to be of clinical relevance, because of its association with prolonged RTP and higher reinjury rate.^20^ Intramuscular tendon involvement is currently included in muscle injury classifications, such as the British Athletic Muscle Injury Classification (BAMIC).^8,18^ Third, our previous studies on MRI at RTP observations were substudies of larger RCTs investigating platelet-rich plasma injection therapy. The participants received saline or platelet-rich plasma injection, which effect on edema and fibrosis on MRI at RTP is unknown.

We conducted this study to overcome these 3 limitations of previous studies with 3.0-T MRI and without an injection intervention to the participants. This observational study aims to describe 3.0-T MRI findings of hamstring muscles in athletes who fully recovered from acute hamstring injury and were cleared for RTP. We hypothesize that the presence of increased signal intensity on fluid-sensitive sequences on MRI (edema) and low-signal intensity suggestive of fibrosis is still observed in RTP athletes after hamstring injury.

## MATERIALS AND METHODS

### Participants

This observational study describing 3.0-T MRI findings after acute hamstring injuries is a substudy from the prospective cohort study of the application of diffusion tensor imaging (DTI) for hamstring injury. The main aim of this study was to evaluate the ability of DTI to detect hamstring muscle injury and its correlation with the convalescent period and return to play. Approval of the study was obtained from Medical Ethics Review Committee Amsterdam University Medical Center (UMC), the Netherlands (METC 2016_033), and registered at Central Committee on Research Involving Human Subjects (CCMO NL55671.018.16).

Potential participants with an acute hamstring injury contacted the coordinating researcher to schedule an appointment at the Amsterdam UMC, The Netherlands. We checked the participants eligibility and obtained the informed consent from the participant who agreed to participate. The sports physician and/or qualified medical team member provided detailed study procedure before they performed history taking and physical examination. The eligibility criteria are presented in Table 1.

**TABLE 1.** Eligibility Criteria

Inclusion	Exclusion
Age > 16 yr old	Complete proximal tendon avulsions (grade 3 hamstring injury)
Acute hamstring injury (<7 d)	Chronic hamstring injury (>2 mo)
Clinical diagnosis of an acute hamstring injury	The injury was caused by extrinsic trauma on the posterior thigh
Posterior thigh pain on anamnestic	Contraindications for MRI (pacemaker, pregnancy, claustrophobia)
Localized pain during palpation of hamstring muscle	Other concurrent injuries inhibit rehabilitation
Localized pain during passive straight leg raises	
Increasing pain during isometric contraction	
For this substudy	
MRI-positive injuries at baseline	
MRI at RTP available (within 10 d of RTP)	

**Figure 1.**
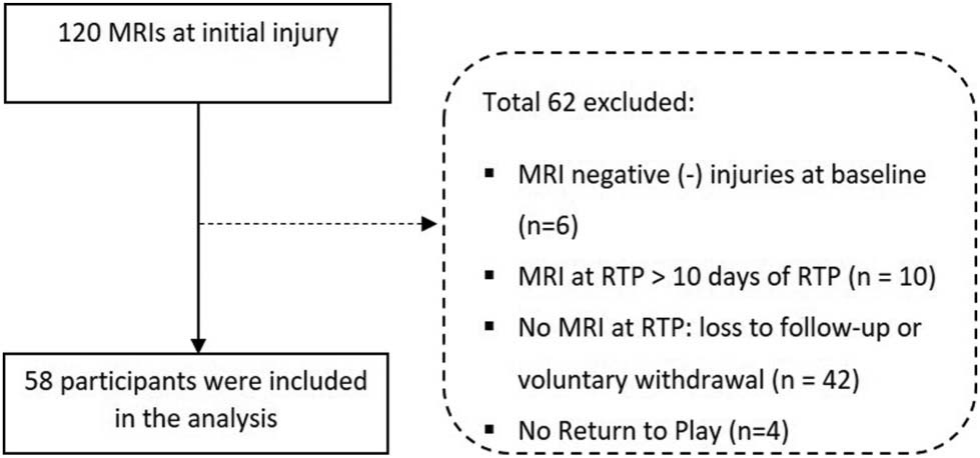
Flowchart of the inclusion process.

In this substudy, we only included participants with positive MRI at initial injury and MRI at RTP available within 10 days of RTP. The flowchart of the inclusion process is presented in Figure 1. We obtained age, gender, sports, level of sports, and side of hamstring injury conducted during baseline examination. We provided examples of rehabilitation programs through our website (www.hamstringonderzoek.nl/) to the participants.

### Magnetic Resonance Imaging

In this study, we analyzed baseline (within 7 days from initial injury) and RTP (within 10 days of RTP) MRIs.

#### Magnetic Resonance Imaging Protocol

The MRI was obtained of both hamstring muscles (injured and healthy side) with a 3.0-T MRI scanner (Ingenia, Philips, Best, the Netherlands) using anterior 16-channel receive coils and posterior 12-channel receive coils in the scanner table. The participants were examined in supine position. The injured hamstring was always placed on the same side in the scanner to avoid B0 and B1 inhomogeneities. The geometrical planning of each scanning session was stored to match RTP with baseline MRI by using anatomical landmarks. Coronal T2 sequence (repetition time/echo time (TR/TE) of 2000/60 ms, the field of view (FOV) of 450 × 450 mm^2^ was obtained. Axial DTI sequence (TR/TE of 5914/54.56 ms, FOV of 480 × 252 mm^2^), Axial T2 sequence (TR/TE of 2000/70 ms, FOV of 480 × 252 mm^2^), Axial Multiple Spin Echo (MSE) sequence for quantitative water T2 mapping (TR/TE of 3000/n*8.0 ms, FOV 480 X252 mm^2^), and Axial Proton Density (PD) Dixon sequence for anatomical reference were obtained.

#### Magnetic Resonance Imaging Assessment

**Figure 2.**
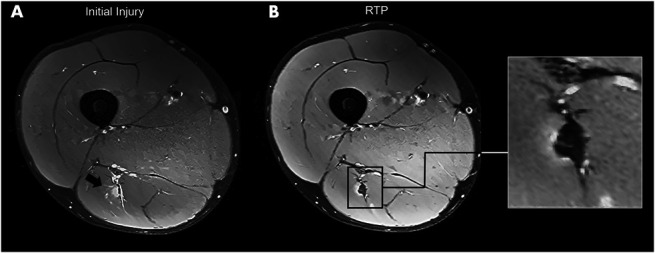
MRI at initial injury and at RTP of biceps femoris long head (BFlh) from a similar participant (A) Axial proton density (PD) Dixon MRI of the initial injury showing presence of edema (arrow) (B) Axial PD Dixon MRI at RTP showing the progression of fibrosis (arrow) with presence of edema.

The MRI was assessed by 1 of 3 musculoskeletal radiologists (F.F.S., S.B., M.M.) with more than 6 years of experience. The same radiologist analyzed the MRI examinations of 1 participant at each different time point to avoid bias. The standardized scoring form from our previous study was used, which showed excellent inter- and intraobserver reliability.^19,22^ We assessed the involved structures (biceps femoris long head [BFlh], biceps femoris short head [BFsh], semimembranosus [SM], and semitendinosus [ST]). Increased signal intensity (edema) defined as an abnormal increased signal in intramuscular compared with the unaffected surrounding muscle tissue (Figure 2A).^19^ Extent of edema was recorded in craniocaudal (cm), anteroposterior (cm), and transverse (cm). The injury was graded according to the modified Peetrons classification^12^ (grade 0: no abnormalities on MRI; grade 1: edema without architectural distortion; grade 2: edema with architectural disruption; grade 3: complete free tendon tear). In addition, we performed BAMIC classification in this study. The injuries are graded 0 to 4 based on MRI features, with grades 1 to 4 including an additional suffix (a: myofascial; b: musculotendinous; c: intratendinous).^14,23^ Low signal (fibrosis) was defined as an abnormally low signal in intramuscular compared with the surrounding muscle tissue (Figure 2B).^19^ We measured the presence of fibrosis with the involved muscles (BFlh, BFsh, SM, ST).

**Figure 3.**
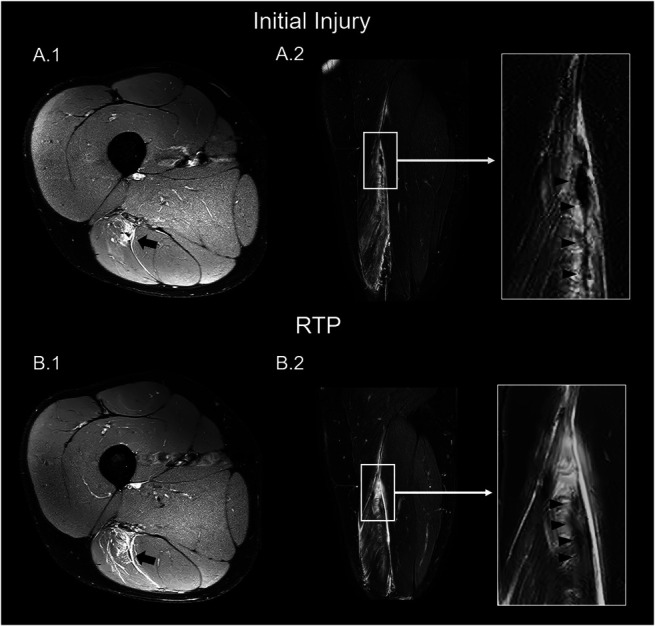
MRI at initial injury (A) and at RTP (B) of BFlh from a similar participant (A.1) The axial PD Dixon MRI at initial injury showing partial thickness intramuscular tendon disruption (arrow) (A.2). The coronal PD Dixon MRI at initial injury showing the waviness and the tendon thickening (head arrow). (B1) The axial PD Dixon MRI at RTP showing partial thickness intramuscular tendon disruption (arrow). (B2) The coronal PD Dixon MRI at RTP showing the waviness and the tendon thickening (head arrow).

Intramuscular tendon disruption was defined as the defect of the section of the tendon that extends along and into the muscle, characterized by loss of low-signal intensity.^24^ We recorded the presence of intramuscular tendon disruption (subdivided into <50%, 50%–99%, and 100% of tendon cross-sectional area [CSA]), tendon thickening, and waviness (Figure 3).^24^ MRI at RTP was compared in the presence of edema, fibrosis, and intramuscular tendon disruption between reinjury and nonreinjury participants.

### Return to Play

Return to play was defined as “the moment a player returns to full, unrestricted training or match.”^25,26^ The clearance of RTP was made by the physiotherapist, medical staff, or the participants themselves on successful completion of the rehabilitation program.

### Reinjury

Hamstring reinjury was defined as an acute onset posterior thigh pain occurring during competition or training in the same leg as the index injury within 1 year after RTP.^24^ The participants were instructed to contact the research coordinator in the event of suspected hamstring reinjury. We also recorded by telephone calls or email periodically to all participants to obtain information about hamstring reinjury within 2 and 12 months.

### Analysis

Statistical analysis was conducted using SPSS version 25.0 (SPSS Inc., Chicago, IL). Descriptive statistics were used to analyze the frequency of extent of edema, involved muscle, MRI grade, presence of fibrosis, and intramuscular tendon abnormalities. A normality test was performed using Kolmogorov–Smirnov test, and data were considered normally distributed if *P* > 0.05. The descriptive data were presented as mean (±SD) or median (IQR) as appropriate for continuous variables and as frequency (%) for categorical variables. The dependent *t* test or Wilcoxon test as a nonparametric test was used to analyze the differences in the extent of edema. We set the significant level at *P* < 0.05.

## RESULTS

Between October 2016 and April 2022, we included 58 participants (Table 2).

**TABLE 2.** Baseline Demographic Characteristics

1	Age (yr ± SD)	27.6 ± 9.4
2	Gender (male/female)	56/2
3	Frequency of sports participation	
	>5 times per week	32 (55%)
	3–4 times per week	5 (9%)
	1–2 times per week	5 (9%)
	Not reported	16 (27%)
4	Sports	
	Football	44 (76%)
	Athletics	3 (5%)
	Field Hockey	5 (8%)
	Rugby	4 (7%)
	Cricket	1 (2%)
	Volleyball	1 (2%)
5	Level of sports	
	Professional	43 (74%)
	Competitive	14 (24%)
	Recreational	1 (2%)
6	Side of injury	
	Right	29 (50%)
	Left	29 (50%)

### Magnetic Resonance Imaging Findings

The characteristics of intramuscular increased signal intensity (edema) on MRI of the initial injury and at RTP are presented in Table 3. Edema is present in 55 (95%) MRIs at RTP and significantly reduced in craniocaudal, anteroposterior, and transverse length compared with initial injury (*P* < 0.05).

**TABLE 3.** MRI Characteristics on Intramuscular Increased Signal Intensity (Edema) of the Initial Injury and at RTP

No	Parameters	Initial Injury (n = 58)		RTP (n = 58)	
1	Intramuscular increased signal intensity (edema)				
	Present	58/58	100%	55/58	95%
	Absent	0/58	0%	3/58	5%
2	Involved muscle				
	BFlh	32/58	55%	31/55	56%
	BFsh	0/58	0%	1/55	2%
	ST	0/58	0%	0/55	0%
	SM	13/58	23%	8/55	15%
	BFlh + BFsh	2/58	3%	2/55	4%
	BFlh + ST	11/58	19%	14/55	25%
3	Grades (modified Peetrons)^12^				
	Grade 0	0/58	0%	3/58	5%
	Grade 1	15/58	26%	27/58	47%
	Grade 2	43/58	74%	28/58	48%
4	Grades (BAMIC)^14^				
	0A	0/58	0%	3/58	5%
	1A	2/58	3%	13/58	22%
	1B	8/58	14%	11/58	19%
	2A	6/58	10%	9/58	16%
	2B	2/58	3%	6/58	10%
	2C	10/58	17%	5/58	9%
	3A	2/58	3%	1/58	2%
	3B	18/58	31%	7/58	12%
	3C	10/58	17%	3/58	5%
5	Length of edema				
	Mean craniocaudal length (cm, IQR)	14.9	0-43	7.4	0-32*
	Median anteroposterior length (cm, IQR)	2.3	0-7	0.9	0-5*
	Median transverse length (cm, IQR)	2.4	0-7	0.9	0-4*

* Statistically significant difference between initial injury and RTP. *P* < 0.05.

BFlh, biceps femoris long head; BFsh, biceps femoris short head; ST, semitendinosus; SM, semimembranosus; IQR, interquartile range.

There was an overall reduction of injury grades both in modified Peetrons and in BAMIC classification. The percentage distribution of injury grade based on modified Peetron and BAMIC is presented in Table 3 and visualized in the online **Supplementary Appendix** (see **Fig. S1**, http://links.lww.com/JSM/A466 and **Fig. S2**, http://links.lww.com/JSM/A467).

The characteristics of intramuscular abnormal low-signal intensity on MRI of the initial injury and at RTP are presented in Table 4. Fibrosis was present in 44 (76%) of the MRIs at RTP.

**TABLE 4.** Characteristics of Intramuscular Abnormal Low-Signal Intensity on MRI (Suggestive for Fibrosis) of Initial Injury and RTP

No		Initial Injury		RTP	
1	Intramuscular fibrosis				
	Present	4/58	7%	44/58	76%
	Absent	54/58	93%	14/58	24%
2	Involved muscles				
	BFlh	2/4	50%	31/44	71%
	ST	0/4	0%	1/44	2%
	SM	0/4	0%	5/44	11%
	BFlh + ST	1/4	25%	6/44	14%
	BFlh + ST + SM	1/4	25%	1/44	2%

BFlh, biceps femoris long head; BFsh, biceps femoris short head; ST, semitendinosus; SM, semimembranosus.

The characteristics of intramuscular tendon disruption on MRI are presented in Table 5. Forty-eight (83%) hamstring injuries involved the disruption of the intramuscular tendon on MRI at initial injury. The percentage of intramuscular thickness discontinuity reduced by almost half from the initial injury to RTP: 79% to 36% (partial thickness discontinuity) and 4% to 2% (complete thickness discontinuity). The tendon thickening and waviness are found at MRIs at RTP by 36 (62%) and 15 (26%), respectively. **Supplemental Digital Content** (see **Table 1**, http://links.lww.com/JSM/A468) shows the comparison between injury grading at initial injury, MRI abnormalities at RTP, and time to RTP for each participant.

**TABLE 5.** MRI Characteristics of Intramuscular Tendon Disruption at the Initial Injury and RTP

		Initial Injury	RTP
1	Intramuscular Tendon Disruption		
	No intramuscular tendon affected	10/58 (17%)	36/58 (62%)
	Partial thickness discontinuity	46/58 (79%)	21/58 (36%)
	<50% of tendon CSA	28/58 (48%)	16/58 (27%)
	50%–99% of tendon CSA	18/58 (31%)	5/58 (9%)
	Complete thickness discontinuity (100%)	2/58 (4%)	1/58 (2%)
2	Involved tendon		
	BFlh	29/48 (60%)	16/22 (73%)
	SM	7/48 (15%)	4/22 (18%)
	ST	2/48 (4%)	0/22 (0%)
	BFlh + ST	10/48 (21%)	2/22 (9%)
3	Waviness	40/58 (69%)	15/58 (26%)
4	Tendon thickening	25/58 (43%)	36/58 (62%)

BFlh, biceps femoris long head; BFsh, biceps femoris short head; ST, semitendinosus; SM, semimembranosus; CSA, cross-sectional area.

### Reinjury

We recorded 3 reinjuries (5%) within 1 year. The presence of edema, fibrosis, and intramuscular tendon disruption on MRI at RTP of participants with reinjuries compared with participants without reinjuries is presented in Table 6.

**TABLE 6.** Edema, Fibrosis, and Intramuscular Tendon Disruption on MRI at RTP of Participants Without Reinjury Compared With Participants With Reinjury Within 1 Year After RTP

	No Reinjury (n = 55)	Reinjury (n = 3)
Presence of edema	51/55 (93%)	3/3 (100%)
The extent of edema mean longitudinal length	5 cm (0-32.3)	12 cm ± 9.7
Presence of fibrosis	40/55 (72%)	3/3 (100%)
Intramuscular tendon disruption (partial and complete thickness discontinuity)	20/55 (36%)	2/3 (67%)
Tendon waviness	13/55 (24%)	2/3 (67%)
Tendon thickening	34/55 (62%)	2/3 (67%)

## DISCUSSION

This study provides a detailed description of the 3.0-T MRI findings at RTP after acute hamstring injuries and is the first 3.0-T MRI study on this topic. The main findings of this study are that at RTP, intramuscular increased signal intensity (edema) was present in more than 9 out of 10 participants (95%), and low-signal intensity (fibrosis) was observed in 7 out of 10 (76%) participants. The intramuscular tendon disruption (partial or complete thickness discontinuity) was recorded at RTP in 4 out of 10 participants (38%), with 6 out of 10 participants having tendon thickening (62%).

Compared with previous studies on 1.5-T MRI, these high percentages of edema at RTP are consistent and comparable with those of our previous study (89%)^19^ and Silder et al (100%),^17^ but are higher than those of Sanfilippo et al^18^ (20.4%). Almost all (95%) of participants showed edema on MRI at RTP. Theoretically, edema is one of the normal inflammatory responses after an acute muscle injury.^27,28^ As the healing process continues, edema will decrease, but mild peritendinous edema identification on MRI findings could also be considered as reparative edema.^28^ Sanfillipo et al reported that residual edema at RTS fully resolved during the subsequent 6 months.^18^

The percentage of low-signal intensity, suggesting fibrosis at RTP in this study, was approximately 1.5 times higher than in our previous study (76% vs 42%).^19^ There are some possible explanations for these findings. First, the 3.0-T MRI might be more sensitive, resulting in a higher percentage of fibrosis. Second, it might be caused by the different study populations. Most participants in this study were professional athletes (74%) with higher modified Peetrons gradings during their first visit (74% injury grade 2 vs 49% the previous studies^19^). The higher modified Peetrons grading may cause the MRI abnormalities finding to take longer time to resolve. Third, the radiologist might be more aware in detecting low-signal intensity. There were 3 reinjuries (5%) within 12 months after RTP.

Intramuscular abnormal low-signal intensity on MRI (suggestive for fibrosis) is a common finding after recovery from hamstring injury. Fibrosis has been suggested as a risk factor for reinjury, although there is lack of evidence from clinical studies.^20^ In the muscle injury healing process, the extracellular matrix transforms into a fibrotic scar. This fibrotic tissue will potentially restrict the regeneration of myofibers and axons across the injury gap by mechanical barrier and decrease the elasticity of the native muscle, which is suggested to increase risk reinjury.^29^ However, fibrosis formation, especially in the initial phase, was also an essential part of the stability of the repair stage.^30,31^ The fibrotic tissue initially formed during the acute healing process will diminish over time.^24^ Reurink showed fibrosis at RTP was not associated with increased reinjury risk at 1-year follow-up after the initial injury.^20^ Our current findings with significantly higher fibrosis rates at RTP on 3.0-T MRI (7 out of 10 participants) justify that the potential association with increased reinjury risk should be reevaluated.

Studies showed that intramuscular tendon involvement in hamstring injury might be a potential risk factor for prolonged RTP and reinjury,^32–34^ although other studies have shown the limited clinical significance to RTP and was not associated with an increased reinjury rate.^23,24,35^

The identification of intramuscular tendon injuries was considered to be important in hamstring diagnosis and rehabilitation management. We used the BAMIC grading system to acknowledge the intramuscular tendon hamstring injury (class “c”), and found a high number of intramuscular tendon disruptions on MRI at the initial injury compared with the previous study.^24^ It might reflect a higher number of participants with intramuscular tendon injury involved in this study. Vermeulen reported that intramuscular tendon discontinuities at baseline demonstrated a sign of healing at RTP.^36^ The study also concluded that complete resolution of an intramuscular tendon injury on MRI is not necessary for successful RTP.^36^ Similar to this study, more than half of the tendon discontinuities at initial injury are reduced in number at RTP.

The high reinjury risk makes RTP decisions for hamstring injury challenging. Currently, there is no strong evidence to associate MRI findings with reinjury. van Heumen et al^37^ showed moderate evidence of intratendinous injuries and biceps femoris injuries with a higher reinjury risk. More recent studies have shown that MRI before RTP may be potentially beneficial in identifying potential reinjury risk factors such as connective tissue gap, loss of tendon tension, intermuscular edema, callus gap, and interstitial feather edema.^38^

This is the first study on MRI observation at RTP after hamstring injury using 3.0-T MRI. The higher Tesla is expected to be more sensitive in detecting abnormality compared with the previous 1.5-T, which is potentially reflected by the higher percentage of fibrosis at RTP. Another strength is that this study is not part of an interventional study, which might influence the findings on MRI at RTP (ie, RCT of Silder et al^17^ and Sanfilippo et al^18^ with 2 different rehabilitation protocols or Reurink^19^ and Van de Made^23^ on platelet rich plasma therapy study). The results of this observational study without intervention can be generalized into the real situation of the athlete at the various levels of competition.

There are some limitations. Most participants in this study were professional male football players, which restricts generalization to other populations. For evaluating a potential association with reinjuries, the number of reinjuries was too low to perform proper statistical analysis. Future studies with a sufficient number of reinjuries should assess the association of several MRI variables as risk factors with the reinjury incidence.

In summary, we reported the 3.0-T MRI findings of 58 participants after an acute hamstring injury who were cleared for RTP. MRI at RTP showed that edema was still present in 9 out of 10 participants and fibrosis in 7 out of 10 participants. Intramuscular tendon disruption was recorded in 4 out of 10 participants, with waviness and tendon thickening present in 3 and 6 out of 10 participants, respectively.

The findings in this 3.0-T MRI showed a higher percentage of MRI abnormalities (edema, fibrosis, and intramuscular tendon disruption) than the previous 1.5-T MRI studies. These results support our previous findings on follow-up 1.5-T MRI after the acute hamstring injury that abnormalities on MRI findings still remain at RTP but its possible association with reinjury risk has to be determined. We conclude that complete normalization of 3.0-T MRI is not expected for RTP decision after a hamstring injury.

## Supplementary Material
