# Supplementary material for: 3.0-Tesla MRI Observation at Return to Play After Hamstring Injuries
Source: Clin J Sport Med. 2024 Nov 20;35(2):119–26. doi: 10.1097/JSM.0000000000001289 (PMC11837960; doi:10.1097/JSM.0000000000001289)

Suppl Fig.1

Figure S1. Percentage distribution of injury grade based on Modified Peetrons at initial injury and at Return to Play

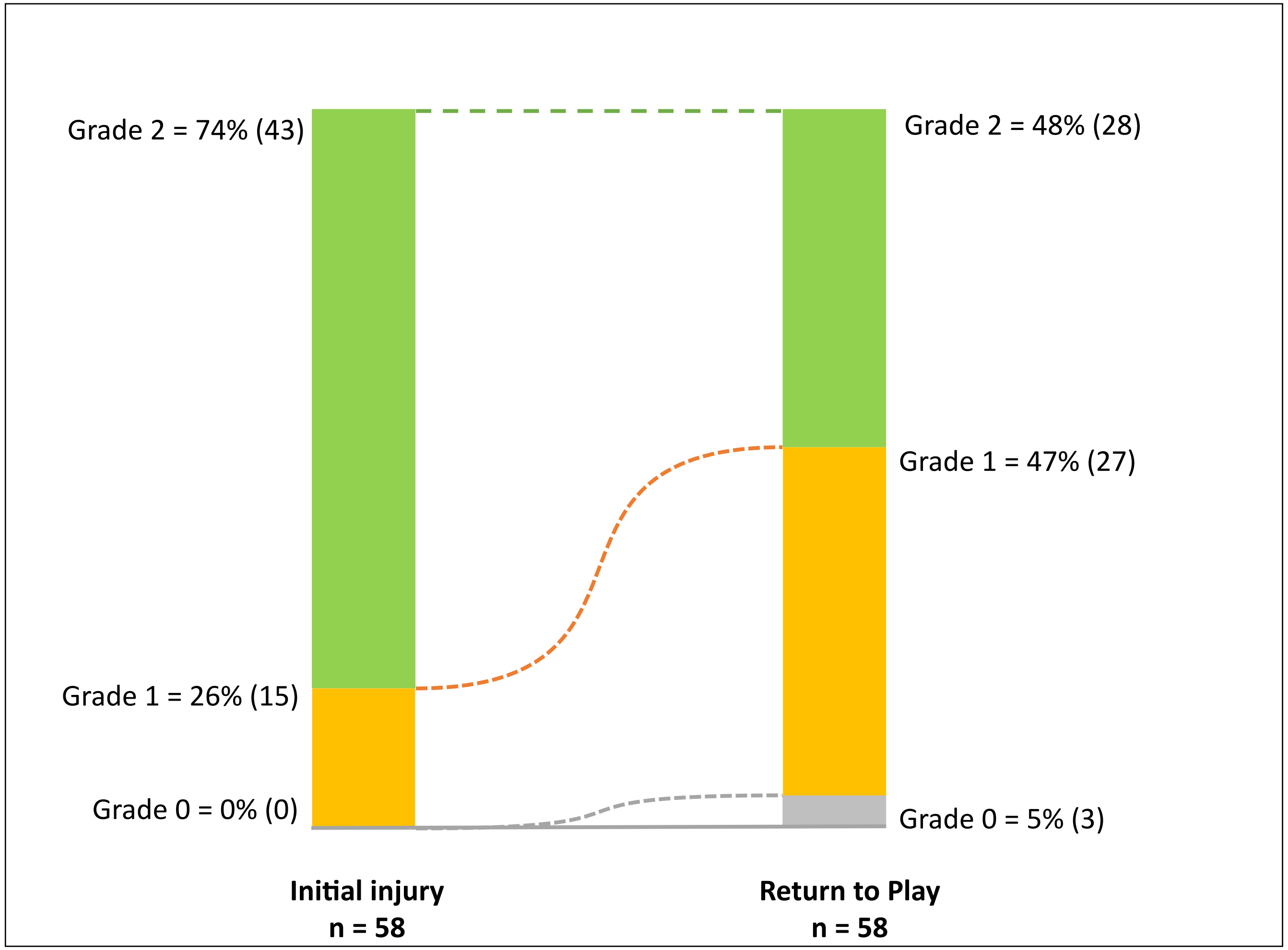

Supplement: Supplementary file 1 [file cjsm-35-119-s001.pdf]
