# Supplementary material for: 3.0-Tesla MRI Observation at Return to Play After Hamstring Injuries
Source: Clin J Sport Med. 2024 Nov 20;35(2):119–26. doi: 10.1097/JSM.0000000000001289 (PMC11837960; doi:10.1097/JSM.0000000000001289)
Supplement: Supplementary file 2 [file cjsm-35-119-s002.pptx]

## Slide 1
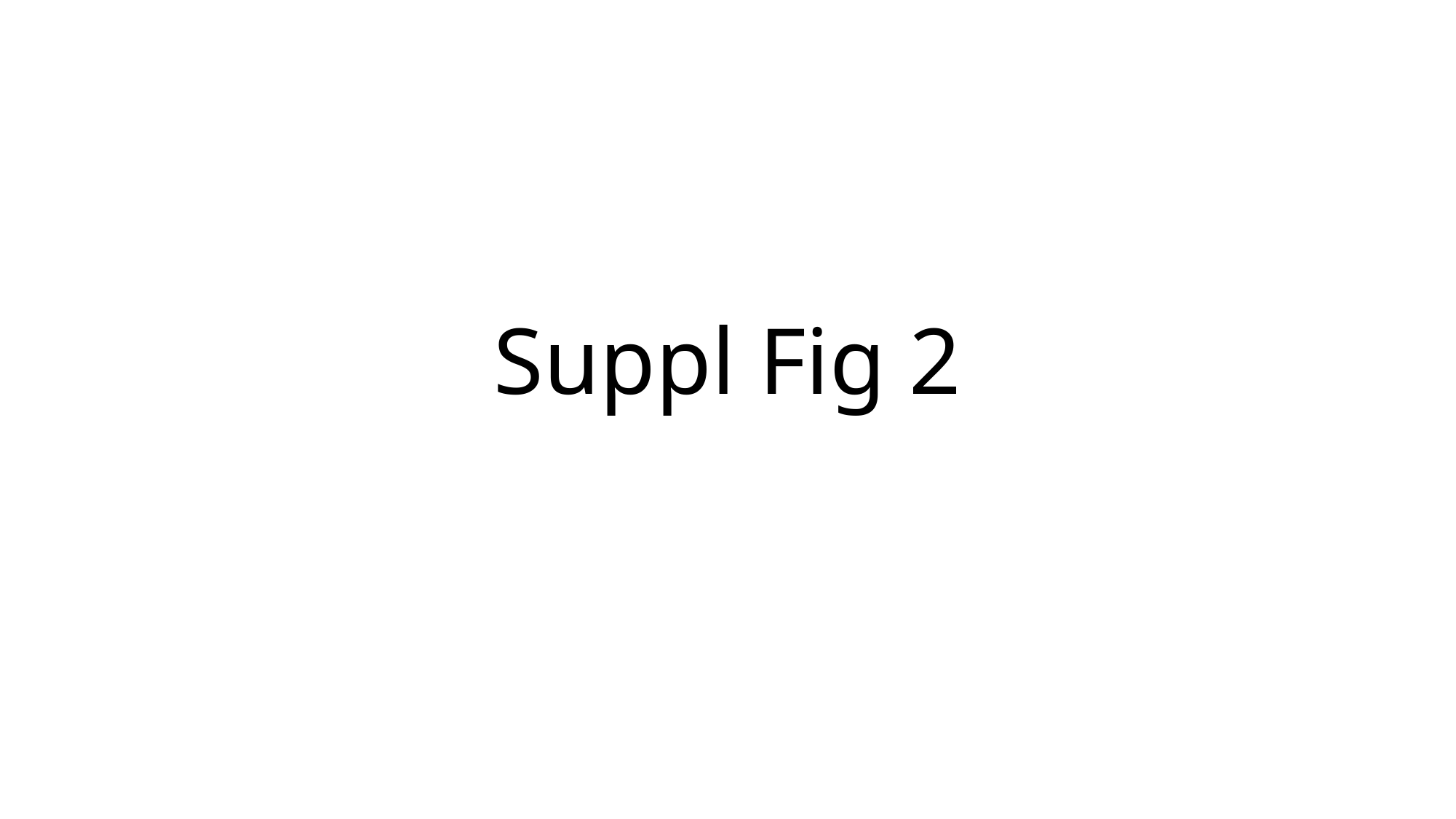

# Suppl Fig 2

## Slide 2
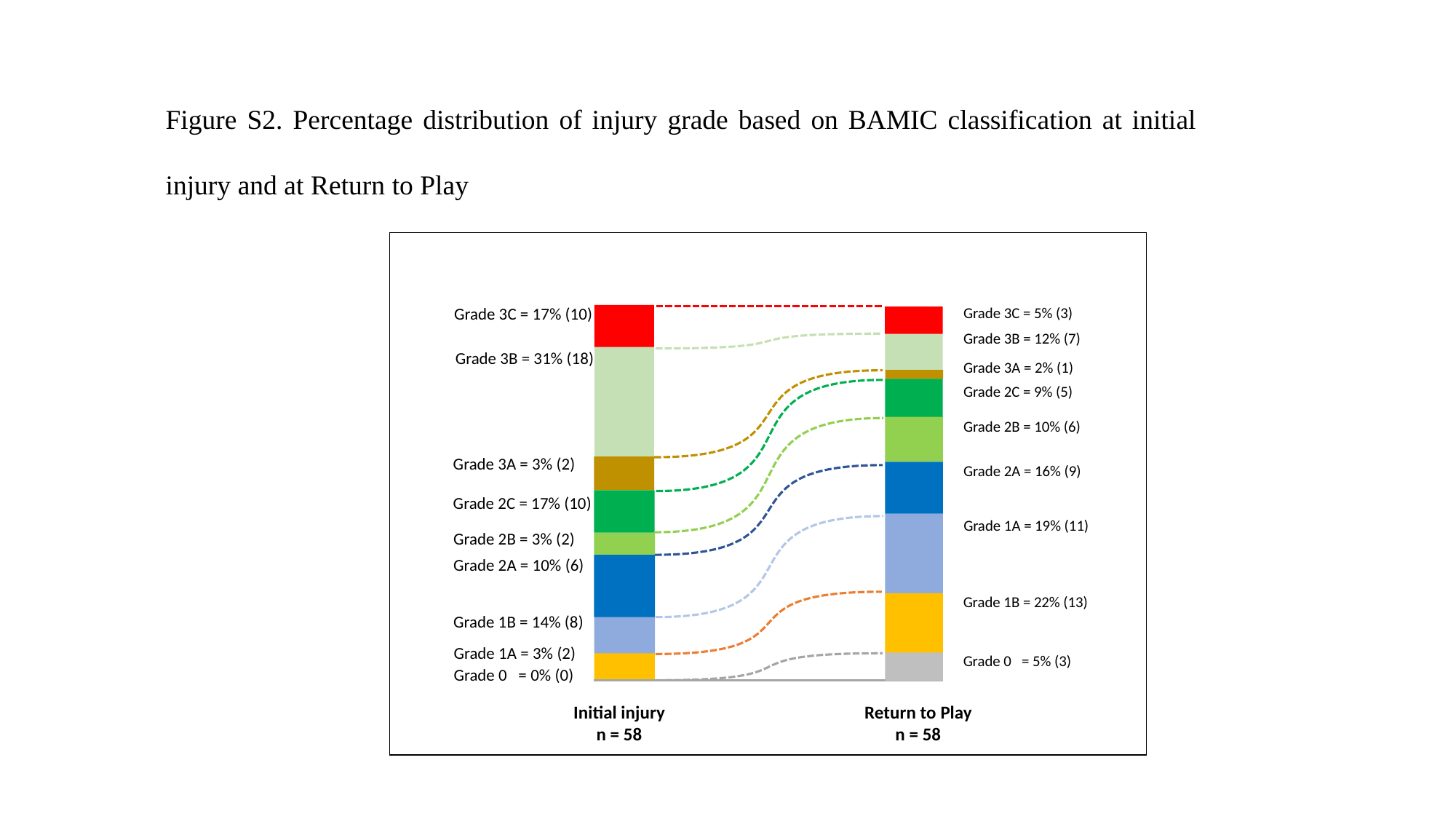

Figure S2. Percentage distribution of injury grade based on BAMIC classification at initial injury and at Return to Play
Grade 3C = 17% (10)
Grade 3C = 5% (3)
Grade 3B = 12% (7)
Grade 3B = 31% (18)
Grade 3A = 2% (1)
Grade 2C = 9% (5)
Grade 2B = 10% (6)
Grade 3A = 3% (2)
Grade 2A = 16% (9)
Grade 2C = 17% (10)
Grade 1A = 19% (11)
Grade 2B = 3% (2)
Grade 2A = 10% (6)
Grade 1B = 22% (13)
Grade 1B = 14% (8)
Grade 1A = 3% (2)
Grade 0 = 5% (3)
Grade 0 = 0% (0)
Return to Play
n = 58
Initial injury
n = 58
