# Supplementary material for: 3.0-Tesla MRI Observation at Return to Play After Hamstring Injuries
Source: Clin J Sport Med. 2024 Nov 20;35(2):119–26. doi: 10.1097/JSM.0000000000001289 (PMC11837960; doi:10.1097/JSM.0000000000001289)
Supplement: Supplementary file 3 [file cjsm-35-119-s003.docx]

Supplementary Table 1. Comparison between injury grading, MRI findings and time to return to play.

| Participants  number | MRI Injury grading at initial Injury  (modifed Peetrons) | MRI at RTP | | | | | Time to RTP (days) |
| --- | --- | --- | --- | --- | --- | --- | --- |
|  |  | **Injury grading at RTP**  **(modified Peetrons)** | **Fibrosis** | **IMT disruption** | **Tendon waviness** | **Tendon thickening** |  |
| 1 | 2 | 2 | + | + | - | + | 15 |
| 2 | 2 | 1 | + | - | - | - | 72 |
| 3 | 1 | 1 | + | + | - | + | 21 |
| 4 | 1 | 1 | + | + | - | + | 54 |
| 5 | 1 | 1 | - | - | - | - | 14 |
| 6 | 2 | 2 | + | + | - | + | 27 |
| 7 | 1 | 1 | + | - | - | - | 25 |
| 8 | 2 | 2 | + | + | + | + | 17 |
| 9 | 2 | 1 | + | + | - | + | 62 |
| 10 | 2 | 1 | + | - | - | - | 53 |
| 11 | 2 | 1 | + | - | - | - | 17 |
| 12 | 1 | 1 | + | - | - | - | 37 |
| 13 | 2 | 1 | + | + | + | + | 42 |
| 14 | 2 | 1 | + | + | + | + | 73 |
| 15 | 2 | 2 | + | + | + | + | 17 |
| 16 | 2 | 1 | + | - | - | - | 20 |
| 17 | 2 | 1 | + | - | - | - | 58 |
| 18 | 2 | 1 | + | - | - | - | 31 |
| 19 | 2 | 1 | + | - | - | - | 15 |
| 20 | 2 | 1 | + | - | - | - | 66 |
| 21 | 2 | 0 | + | - | - | - | 60 |
| 22 | 2 | 1 | + | - | - | - | 45 |
| 23 | 2 | 1 | + | - | - | - | 45 |
| 24 | 1 | 1 | - | - | - | - | 82 |
| 25 | 2 | 2 | + | + | + | + | 9 |
| 26 | 2 | 0 | - | - | - | + | 105 |
| 27 | 2 | 2 | + | + | - | - | 31 |
| 28 | 2 | 2 | - | + | - | + | 124 |
| 29 | 1 | 1 | - | - | - | + | 18 |
| 30 | 2 | 2 | + | + | + | + | 44 |
| 31 | 2 | 2 | + | - | + | + | 53 |
| 32 | 2 | 2 | + | - | - | + | 38 |
| 33 | 2 | 2 | + | - | - | + | 45 |
| 34 | 2 | 2 | + | - | - | + | 51 |
| 35 | 1 | 1 | - | - | - | - | 23 |
| 36 | 2 | 2 | + | - | - | + | 42 |
| 37 | 2 | 2 | + | - | - | + | 56 |
| 38 | 2 | 2 | + | + | + | + | 39 |
| 39 | 2 | 2 | + | - | + | + | 79 |
| 40 | 1 | 1 | - | - | - | - | 21 |
| 41 | 2 | 0 | - | - | - | - | 82 |
| 42 | 1 | 1 | - | - | - | - | 16 |
| 43 | 1 | 1 | - | - | - | - | 14 |
| 44 | 2 | 2 | - | - | - | + | 25 |
| 45 | 1 | 1 | - | - | - | + | 11 |
| 46 | 2 | 2 | + | + | - | + | 17 |
| 47 | 2 | 2 | + | + | + | + | 11 |
| 48 | 2 | 2 | + | + | + | + | 31 |
| 49 | 2 | 2 | + | - | - | + | 35 |
| 50 | 2 | 2 | + | + | + | + | 37 |
| 51 | 2 | 2 | + | + | - | + | 71 |
| 52 | 1 | 1 | + | - | - | - | 66 |
| 53 | 2 | 2 | + | + | + | + | 46 |
| 54 | 2 | 2 | + | + | + | + | 31 |
| 55 | 2 | 2 | + | + | + | + | 119 |
| 56 | 2 | 2 | - | - | - | + | 39 |
| 57 | 1 | 1 | + | - | - | + | 33 |
| 58 | 2 | 2 | - | + | - | + | 29 |

Abbreviations: RTP = Return to play. IMT = Intramuscular tendon. MRI = Magnetic Resonance Imaging. + = present on MRI. - = not present on MRI.
